# Surgical Results of Intradural Extramedullary Tumors

**DOI:** 10.4055/cios.2009.1.2.74

**Published:** 2009-05-27

**Authors:** Kyung-Won Song, Sung-Il Shin, Jin-Young Lee, Gab-Lae Kim, Yoon-Suk Hyun, Deok-Yong Park

**Affiliations:** Department of Orthopaedic Surgery, Kangdong Sacred Heart Hospital, Hallym University School of Medicine, Seoul, Korea.

**Keywords:** Intradural, Extramedullary, Tumor

## Abstract

**Background:**

To report the treatment results of 12 patients who underwent a total excision of intradural extramedullary tumors.

**Methods:**

Twelve cases of histopathologically confirmed intradural extramedullary tumors were treated surgically between February 2002 and March 2005. There were 8 males and 4 females with an average age of 42.6 years. The mean postoperative follow-up period was 24.2 months. The histopathological findings, locations of the tumors, and clinical results were analyzed. The neurological findings obtained during the preoperative stage and the postoperative follow-up were evaluated according to the Frankel classification.

**Results:**

The histopathological results are as follows: 4 cases of a meningioma, 4 cases of a schwannoma, 2 cases of an epidermoid cyst, 1 case of an arachnoid cyst, and 1 case of an ependymoma. The locations of the tumors were as follows: 7 cases in the thoracic region, 4 cases in the lumbar region, and 1 case in the cervical region. At the final follow-up, a 2-grade and 1-grade improvement was observed in 1 and 7 cases, respectively. There were no changes in the Frankel grade in 4 cases. The preoperative neurological deficit improved within 8 postoperative weeks in most cases and within 1 postoperative year in all cases. Postoperatively, there were 2 cases of cerebrospinal fluid leakage and 2 cases of paresthesia.

**Conclusions:**

Intradural extramedullary tumors detected by MRI are mostly benign and good clinical results can be obtained when treated surgically. Therefore, more active surgical approaches by orthopedic surgeons are recommended to decrease morbidity.

Intradural extramedullary tumors are rare central nervous system tumors that are found only in 0.3 out of 100,000 patients each year. Despite their rarity, there are many reports of these tumors in the literature. Since Gower and Horsley reported satisfactory results for the first time by removing a tumor located at the T6-7 level in a 42-year-old male in 1887, more aggressive treatment approaches aimed at preserving and further enhancing the neurological function have been developed over the past 30 years. In addition, great strides have been made recently in the prognosis of surgery due to the development of diagnostic tools, such as CT and MRI, an understanding of the precise anatomical structures, and the advancement of surgical instrumentation and techniques. Nevertheless, early-stage intradural extramedullary tumors are difficult to detect. They are not easily differentiated from lower lumbar disc diseases, such as herniation of the intervertebral disc and spinal stenosis. These tumors lack obvious clinical symptoms until compression or neurological deficit occurs. However, a delay in surgical removal and treatment can lead to permanent neurological deficits. This study examined 12 patients who were followed for more than 1 year after the diagnosis and underwent a total extirpation of the intradural extramedullary tumor.

## METHODS

### Materials

Of the patients treated surgically for intradural extramedullary tumors between February 2002 and March 2005 at our hospital, 12 patients who showed histopathological evidence and were available for a more than a 1 year follow up were enrolled in this study. The mean age of the patients was 42.6 years (range, 19 to 74 years). There were 8 males and 4 females. The mean postoperative follow-up period was 24.2 months (range, 20 to 56 months). The histopathological findings, locations of the tumor, clinical symptoms, durations of symptoms, and radiological findings were analyzed and MRI was performed in all cases.

### Surgical Technique

The patient was placed in the prone position under general anesthesia. A laminectomy was performed regardless of the location or type of tumor through the posterior approach alone. A longitudinal incision was made in the dura mater and the tumor was detached from the dura mater and removed. When a nerve fiber was attached to a tumor, a nerve stimulator was used to determine if it was a sensory or motor nerve branch. The sensory nerve branch was removed while the motor nerve branch was preserved through careful detachment. Posterior interbody fusion with instrumentation was also performed in two cases where the tumor was so large as to cause posterior instability.

### Postoperative Evaluation

The postoperative evaluation involved a comparison of the neurological findings were graded according the Frankel classification preoperatively and at the last follow-up.

## RESULTS

**Fig. 1:**
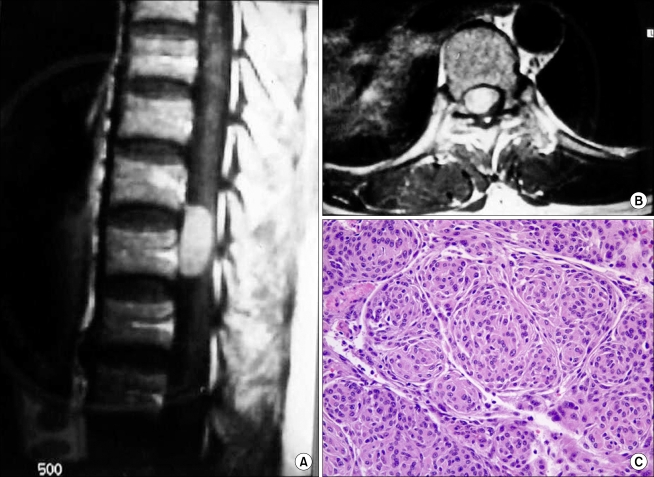
Forty-seven-year-old female with intradural extramedullary menigioma with paraparesis. (A) T1-weighted sagittal MR image shows homogenous high signal intensity mass. The tumor is located at the T10-11 level. (B) T1-weighted coronal MR image shows a space occupying mass compressing the spinal cord. (C) Histopathology findings shows a whorl pattern of cellular growth characteristics of a meningioma (original magnification, × 300).

**Fig. 2:**
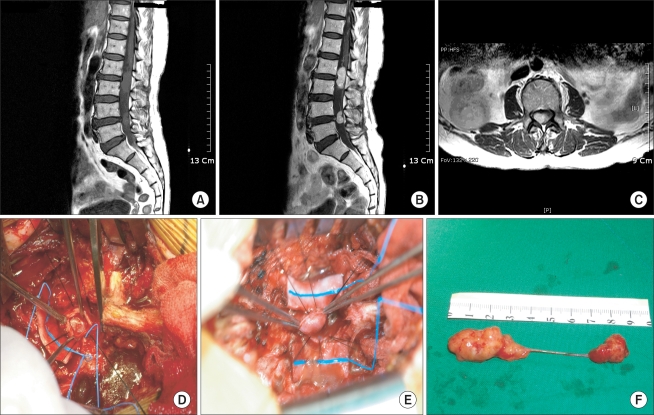
Seventy-four-year-old female with intradural extramedullary schwannoma with back pain and a sensory decrease. (A) T1-weighted sagittal MR image shows two homogenous iso-signal intensity masses. The tumor is located at the L2-3, 4-5 level with dumbbell shape for-mation. (B) Gadolinium enhanced T1-weighted sagittal MR image shows marginal enhancement of heterogeneous high signal intensity mass. (C) Gadolinium enhanced T1-weighted axial MR image shows space occupying intradural extramedullary mass compressing spinal cord to the left side. (D, E) Intra-operative findings of intradural extramedullary mass excision. (F) Extracted tumor showing a well encapsulated masses measuring 3.5 × 1.5 × 1 cm and 1.5 × 1 × 1 cm connected with linear stock.

**Fig. 3:**
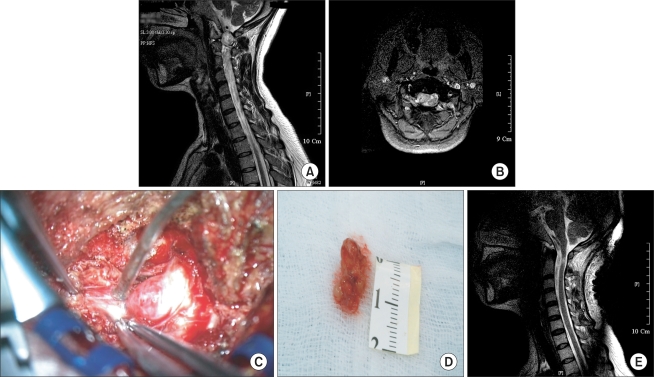
Thirty-five-year-old male with intradural extramedullary schwannoma with both upper & lower extremity weakness and tingling sensation below C3 level. (A) T2-weighted sagittal MR image shows a huge homogenous high signal intensity mass. The tumor is located at the C1-2 level. (B) T2-weighted coronal MR image shows a space occupying mass compressing the spinal cord severely. (C) Intra-operative findings of intradural extramedullary mass excision. (D) Extracted tumor showing a well encapsulated mass measuring 1.6 × 1 × 0.8 cm. (E) Postoperative MR image shows mass lesion removed at the C1-2 level, but the spinal cord signal changes remain.

**Fig. 4:**
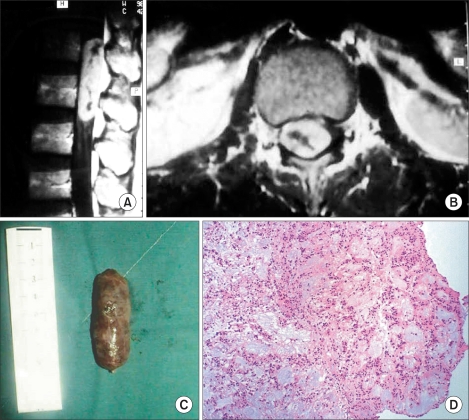
Fifty-seven-year-old male with intradural extramedullary ependymoma with lower leg weakness. (A) T1-weighted sagittal MR image shows heterogeneous high signal intensity mass. The tumor is located at the L2-3 level with longitudinal oval shape formation. (B) T1-weighted coronal MR image shows space occupying mass compressing the spinal cord severely. (C) Extracted tumor showing a well encapsulated capsular shaped mass measuring 4.2 × 1.3 × 1 cm. (D) Histopathology findings shows a circular arrangement of cells around a clear space: perivascular pseudorosette formation characteristics of ependymoma (original magnification, × 60).

Among various symptoms, the major ones at the time of admission included pain in the abdomen and lower limbs, sensory disturbance of the lower limbs in 5 cases, and motor weakness of the lower limbs in 6 cases. Remarkable relationships were found between the symptoms and locations of a tumor. Tumors in the cervical spine were associated with pain in the neck and motor weakness in the distal upper limbs. Thoracic tumors were related to severe pain in the thoracic spine due to compression of the spinal cord by the tumor. Paresis of both lower limbs was also more related to thoracic tumors than to the lumbar ones. The chief complaints of the patients with conus medullaris and cauda equina tumors included bladder and bowel dysfunction and saddle anesthesia. In particular, pain in the lesions of the conus medullaris tended to precede a sphincter disturbance occurring in the late stage. In the physical examination, 6 cases of motor weakness, 5 cases of sensory disturbance, and 4 cases of sphincter disturbance were observed (Tables 1 and 2). The symptoms reported by patients were similar to the ordinary ones found in lower lumbar disc diseases. The mean period from the onset of subjective symptoms to admission was long, 59.4 months (range, 9 to 241 months). The plain radiographs taken at the time of admission revealed no abnormalities in 10 cases and mild kyphoscoliosis in 2 cases. A MRI scan performed at that time showed intradural extramedullary spinal cord compression in all cases. Regarding the location of a tumor, 7 were found in the thoracic spine (58.3%), 4 in the lumbar spine (33.3%), and 1 in the cervical spine. The most common histopathological findings were meningioma (Fig. 1) and schwannoma (Figs. 2 and 3) in 4 cases (33.3%) each. An epidermoid cyst was diagnosed histopathologically in 2 cases, an arachnoid cyst in 1 case, and an ependymoma (Fig. 4) in 1 case. The preoperative Frankel grade was B in 1 case, C in 2 cases, D in 6 cases, and E in 3 cases. During the postoperative follow-up period, 1 and 7 patients showed a 2-grade (8.3%) and 1-grade improvement (58.3%), respectively. Four patients (33.3%) showed no changes. Accordingly, 1 patient fell into the category of C, 2 into D, and 9 into E. No patient deteriorated (Tables 1 and 3). The changes in grade occurred within 2 postoperative months in 5 cases (62.5%) and within 6 to 12 postoperative months in 3 cases (37.5%). The preoperative neurological deficits improved within 8 postoperative weeks in most cases and within 1 year in all cases. With regard to the postoperative complications, cerebrospinal fluid (CSF) leakage was observed in 2 patients and paresthesia in another 2 patients. The former was resolved with the placement of a temporary lumbar drain. The latter, although improved slightly after ≥ 6 postoperative months, was still present.

## DISCUSSION

According to Nittner,1) the incidence of intradural extramedullary tumors is 0.3 out of 100,000 people, accounting for 84% of intradural tumors found in 45% of patients with spinal cord tumors with no gender preference. More than 50% of these tumors are found in the thoracic spine, and they occur in the cervical and lumbosacral spine at a similar rate, 22% and 18%, respectively.1) Histopathological diagnoses include schwannoma in 23-48%, meningioma in 9.6-35%, neurofibroma in 4-23%, and metastatic tumors in 6.4-25% of the total number of cases.^1^,2) Common clinical symptoms in patients with tumors are pain and paresthesia in the abdomen and the lower limbs, motor abnormality, and dysuria.^2^,3) According to Shin et al.,4) while abdominal pain is the first subjective symptom, most patients complain of paresthesia and motor abnormalities at the time of admission. This was attributed to the difficulty in making an early diagnosis because the tumors grow slowly, produce vague symptoms in the early stages, and present with pain and radiating pain similar to those found in lower lumbar disc diseases.5) For these reasons, as was the case in the study by Shin et al.,4) there was a delay from the development of subjective symptoms to admission in this study, an average of 59.4 months in the present study. Therefore, it is believed that spinal cord tumors should also be taken into consideration as a possible diagnosis when a patient complains of symptoms that are usually found in lower lumbar disc diseases, such as intervertebral disc herniation and spinal stenosis.

While some authors reported that pedicle erosion, vertebral body erosion, foraminal widening, neural foramen widening, and scoliosis were found on plain radiographs in approximately 38-56% of patients with an intradural extramedullary tumor,2) normal findings were observed in 10 cases except for 2 cases of kyphoscoliosis. Recently, MRI was regarded as being a helpful tool with regard to tumors: in assessing the size, shape, and anatomical relations with the adjacent structures, particularly with the dura mater and spinal cord; and in determining the basic treatment guidelines and surgical approaches by allowing early detection of a metastatic tumor.6) On MRI, a schwannoma, which is one of the most common primary spinal cord tumors and can be either intradural or edpidural, is observed as a heterogeneous low signal intensity mass (Figs. 2 and 3).7) A meningioma that is an intradural extramedullary and grows slowly into the subarachnoid space8) is identified as a homogeneous low signal intensity mass on the T1-weighted images and as a homogenous high signal intensity mass on the T2-weighted images, and is sometimes calcified (Fig. 1).6)

Many intradural extramedullary tumors are benign and are treated primarily with an aggressive surgical excision because they can be separated easily from the spinal cord due to the developments of diagnostic and surgical instrumentation as well as microsurgical and neuroanesthesia techniques.^9^-12) In this study, as the tumors in most of the 12 patients were surrounded by a capsule with a well-defined margin, they could be easily dissected. During the procedure, posterior lumbar interbody fusion and instrumentation were also performed in two cases where the tumors were so large as to cause posterior instability. The nerve roots that course through the tumor and attached dura mater should also be excised in order to prevent relapse of the tumor.1) Therefore, the intradural tumors as well as the attached dura mater and nerve roots were removed. During this resection, care was taken to remove the sensory nerve branch without damaging the motor nerve branch using the nerve excitability test. However, the sensation of the removed branch was not easily restored. When it comes to tumors, complete surgical removal is the ultimate treatment method and the treatment outcome is dependent on the neurological condition, the extent of the excision, and histopathological findings. Therefore, aggressive approaches are of considerable importance in the diagnosis and the treatment of tumors. Klekamp and Samii13) reported that the overall outcome could be favorable when the interval from the diagnosis prior to the development of severe neurological deficits to surgery was short and improvements in Frankel grade were observed in 8 of the 12 patients.

Intradural extramedullary tumors, which are detected by MRI and tend to be histopathologically benign, can be separated completely from the spinal cord without difficulty by surgery. In addition, good treatment outcomes and prognoses can be expected after surgical removal of intradural extramedullay tumors. Therefore, aggressive surgical approaches for the treatment of intradural extramedullary tumors by orthopedic surgeons are recommended

## Figures and Tables

**Table 1:**
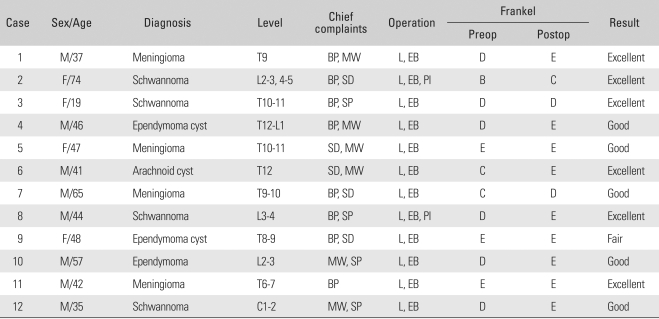
Analysis of the Patients' Data

BP: Black pain, MW: Motor weakness, SD: Sensory decrease, SP: Sphincter disturbance, L: Laminectomy, EB: Excision and biopsy, PI: Posterior instrumentation.

**Table 2:**
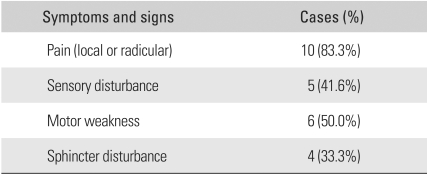
Clinical Symptoms and Signs on the Initial Examination

**Table 3:**
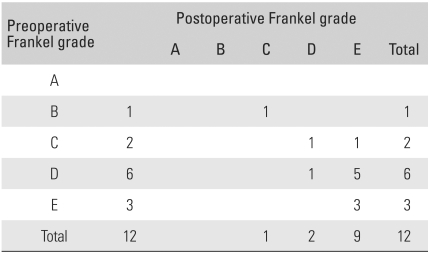
Functional Outcome Using the Frankel Grade
